# Coronavirus questions that will not go away: interrogating urban and socio-spatial implications of COVID-19 measures

**DOI:** 10.35241/emeraldopenres.13561.1

**Published:** 2020-04-16

**Authors:** Ashraf M. Salama

**Affiliations:** 1 Cluster for Research in Architecture and Urbanism of Cities in the Global South (CRAUCGS), Department of Architecture, University of Strathclyde, Glasgow, G11XQ, UK

**Keywords:** Architecture, Coronavirus, COVID-19, Social Distancing, The Actual Normal, The New Normal, Urbanism, Virtual World

## Abstract

The highly contagious coronavirus and the rapid spread of COVID-19 disease have generated a global public health crisis, which is being addressed at various local and global scales through social distancing measures and guidelines. This is coupled with debates about the nature of living and working patterns through intensive utilisation of information and telecommunication technologies, leading to the social and institutional acceptability of these patterns as the ‘new normal.’  The primary objective of this article is to instigate a discourse about the potential contribution of architecture and urban design and planning in generating knowledge that responds to pressing questions about future considerations of post pandemic architecture and urbanism. Methodologically, the discussion is based on a trans-disciplinary framework, which is utilised for conceptual analysis and is operationalized by identifying and discoursing design and planning implications. The article underscores relevant factors; originates insights for areas where future research will be critically needed, through key areas: a) Issues related to urban dynamics are delineated from the perspective of urban and human geography, urban design and planning, and transportation engineering; b) Questions that pertain to socio-spatial implications and urban space/ urban life dialectics stem from the field of environmental psychology; and c) Deliberations about new environments that accommodate new living/working styles supervene from ethnographical and anthropological perspectives.  The article concludes with an outlook that captures key aspects of the needed synergy between architectural and urban education, research, and practice and public health in a post pandemic virtual and global world.

## Introduction: COVID-19 brief account and disciplinary contributions

On 20th January 2020, epidemiologists at the Chinese Centre for Disease Control and Prevention published an article stating that the first cluster of patients with ‘pneumonia of an unknown cause’ had been identified on 21st December 2019 in Wuhan ( BFPG, 2020), a city with a population of more than 11 million. Following this, announcements were made -- that thousands of cases were identified in China and substantial numbers of cases were broadcasted in many countries around the world. On 30th January WHO’s Director-General declared the coronavirus outbreak a public health emergency of international concern ( WHO, 2020). Given that thousands of cases have been reported reaching all corners of the world in one month, this declaration was coupled with a number of recommendations related to early detection of infection, isolating and treating infected cases, contact tracing and social distancing measures that correspond to the level of risk in each country, with a key objective to interrupt or delay and hopefully limit the virus spread.

Within the United Kingdom, Public Health England announced it is moving the risk level to the British public from ‘very low’ to ‘low’ on 22nd January. This is also when first two patients in the UK tested positive for coronavirus after two Chinese nationals from the same family staying at a hotel in York fell ill. A plane clearing Britons from Wuhan and evacuees went into a 14-day quarantine at a specialist hospital in Merseyside ( BFPG, 2020). After confirming the first transmission of disease within the UK, the government decided not to follow Italy and China where the highest figures of infection and death were recorded; the lockdown approach that imposes restrictions on liberty and movement was not favoured by the government. Instead, throughout February 2020 the government advised a range of voluntary restrictions such as ’social distancing’ and, if any symptoms are exhibited, self-isolation and quarantine. On the 5th March, infected cases were reported in all areas of the UK: 105 in England, six in Scotland, three in Wales, and one in Northern Ireland ( BBC, 2020).

By the third week of March, the Prime Minister announced a further set of measures as part of a nationwide lockdown ( Beadsworth, 2020). This was due to the continuous increase in infected and death cases, which reached, according to Public Health England, a total of 14,543 cases and 759 deaths on 27th March ( PHE, 2020). The virus is highly infectious and, at the time of writing this article, there is no known vaccine or specific antiviral treatment for COVID-19 disease, despite claims by various governments that a vaccine or treatment is being developed. The key measure of the global public health campaign in response to the pandemic is social distancing, which, in essence, is avoiding face contact and encouraging physical distancing.

The significant contributions to stop and treat viruses and associated diseases fall within various academic disciplines and professions. Most important, medical scientists, biologists, and public health researchers are the key contributors to a pandemic of this nature and scale. They conduct laboratory research to understand the attributes of the virus and the characteristics of the family of viruses it belongs to. They experiment, develop, test, and advance vaccines for mass use, and eventually identify effective treatments. Professionals and scholars from other disciplines are also contributors, such as mathematicians and computer scientists, whose work, through modelling techniques, enable an effective understanding of global patterns of virus spread and mortality rates. Social and behavioural scientists contribute to the development of policies in the sense that they enable institutions and organisations to identify risks and manage responses as they relate to their employees and communities, while addressing issues related to anxiety, loneliness, and mental health.

Architecture and urbanism as academic disciplines and professions that influence, in many different ways, individuals, communities, and societies, can support efforts through: developing new insights into the impact of a pandemic on cities and urban environments now and in the future; developing new understandings relevant to the characteristics of urban spaces which ensue from these insights; conducting research to comprehend the socio-spatial implications of COVID-19 measures and guidelines introduced by governments and authorities to fight the spread of the disease; identifying new conceptions related to emerging lifestyles which stem from the new spatial environments that integrate working and living patterns; and ultimately developing design responses towards creating healthy environments that successfully accommodate the infected populations while addressing the associated social and psychological ramifications.

At the time of writing this article, there is no sufficient or available empirical research conducted to address to the preceding areas of potential contributions both at the architectural (building) and urban (city) scales. This article nonetheless aims to interrogate these areas by instigating important questions while striving to generate responses through conceptualisation, operationalization, and referencing the available literature. The fundamental aim is to underscore relevant factors, originate insights of potential use to policy makers, architects and planners, highlight areas where future research will be critically needed, and emphasise the positive role architecture and urban design and planning fields can play in developing healthy environments in a globally virtual world.

## Arising questions: social distancing and the acceptability of the new normal

Social distancing measures are a vital part of mitigating pandemics. They complement other measures in decreasing the prospect of the spread of disease. The current body of knowledge points out that social distancing is not a new measure to mitigate the spread but has been introduced and practiced during the past several decades. Researchers have developed important evidence on the potential impact of social distancing, arguing that it is moderately effective ( AHMPPI, 2019). Therefore, where socio-economic impacts are insignificant, social distancing has been viewed, at least, as an acceptable temporary measure. While referred to as physical distancing in many writings, social distancing is a set of infection control actions envisioned to slow down or delay and eventually stop the spread of an infectious disease ( Harris *et al.* 2020). It aims to reduce the likelihood of contact between persons carrying an infection, and others who are not infected, so as to reduce virus transmission, sickness and minimise mortality ( Hensley, 2020).

Despite the suggestion made by AHMPPI (2019), there appears to be growing evidence of the effectiveness of social distancing when infection is transmitted through droplet contact, which may result from all forms of physical contact between people and contact with contaminated surfaces and fabrics. It is also effective when transmission is airborne where the virus can survive in the air for a period of time. There is no clear evidence, however, that social distancing is effective when infection is transmitted through contact with water, food, or through insects.

Social distancing measures were introduced by the World Health Organization in response to the initially gradual and then exponentially global spread of coronavirus. The measures were generic in nature and were subject to various interpretations of governments in the global north and the global south so they can respond to the level of risk in each country or even residential neighbourhood or locality. These interpretations range from flexible or minor social distancing measures to lockdown or, in some countries, various types of curfews during defined timeframes within the day or the week.

Measures of social distancing are practiced at both institutional and individual levels. At an institutional level, there is common agreement on what social distancing entails, as evidenced in government documents of countries around the world ( PHE, 2020; PanCAP, 2020). In this context, the key elements are school closure, workplace closure, and cancellation of mass gatherings. This is further expanded to include closure of small businesses, restaurants, cinemas, theatres, bars, pubs, and clubs. According to PHE (2020), at an individual level, social distancing entails reducing or minimising interaction between people and involves: a) avoidance of non-essential use of public transport especially during rush hours; b) working from home, where possible with regulations set for employers to support this; c) avoidance of contact with someone who is displaying symptoms of coronavirus (COVID-19), which include high temperature and/or new and continuous cough; d) avoidance of large gatherings, and gatherings in smaller public spaces; and e) avoidance of gatherings with friends and family, with recommendations to use distance technology including phone, internet, and social media.

Strikingly, many social media posts, online newspapers, portals and discussion boards, and academic platforms are now introducing and discussing the notion of the ‘new normal’, portraying this as a new paradigm which will involve many new realities and intensive online activities that range from retail and shopping to banking and higher education prevision, to name a few. It is argued, “social distancing is here to stay for much more than a few weeks. It will upend our way of life, in some ways forever” ( Lichfield, 2020). There is strong evidence that there is now a high degree of acceptability among governments, institutions, organisations, and universities that contemporary societies are approaching a new era, characterised by intensive digital/virtual practices, to which the way of life must be adapted. In particular and in the immediate future until it becomes ‘actually normal,’ there is a general appreciation that such a new normal will have negative consequences, which include loneliness, reduced productivity, unhealthy sleeping and eating habits, potential obesity, and loss of various benefits associated with reduced human-human and human-environment interactions.

With social distancing measures, the remarkable shift from the physical to the virtual and the acceptance of this, there are many social and spatial implications that architects, planners, and built environment professionals would be keen to examine. In this respect, key questions arise to address various scales and scopes and can be outlined as follows:

- *What is the nature of transformations in urban dynamics post pandemic?*
- *What are the key socio-spatial implications of distancing measures?*
- *Could COVID-19 alter the understanding of urban space and urban life dialectics? And would engagement with nature be favoured over human-human / human-built environment engagement?*
- *Would post-pandemic epoch generate new environments that accommodate new living/working styles?*

The preceding questions do not cover the entire spectrum of issues and potential impacts arising from social distancing and the new normal. For example, issues associated with risk management, construction processes and practice management are not included. Yet, the questions cover some fundamentals that are believed to be of interest to the global community of architects and urban designers and planners, and that are predicted to generate new conceptions, develop new insights, and eventually inform new thinking about the future of architecture and urbanism. This would also instigate a discourse on role of architecture and urbanism in developing healthy environments and supporting emerging lifestyles in a post pandemic virtual era.

## Initial response: a trans-disciplinary framework for analysis

**Figure 1.**
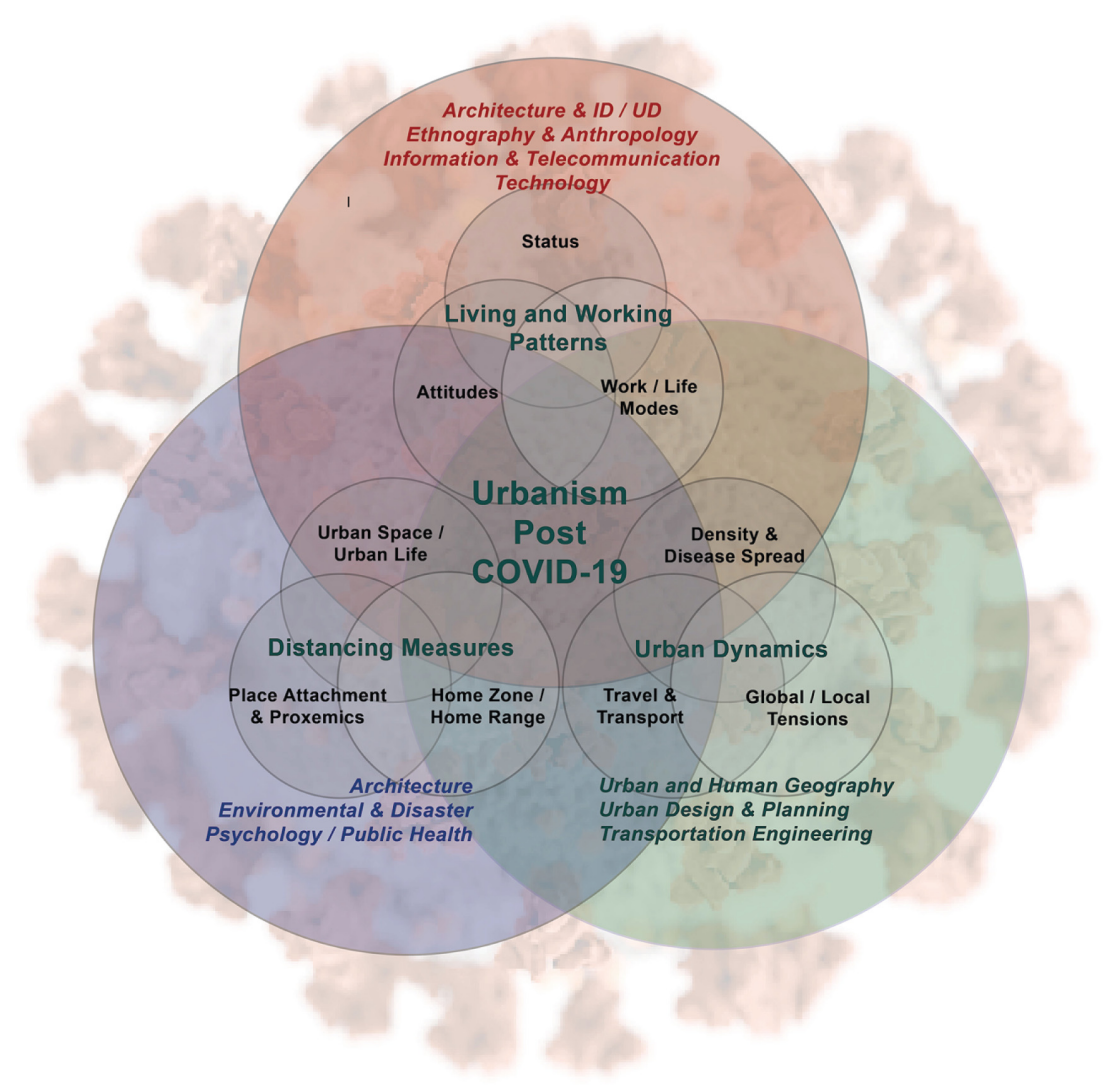
A conceptual trans-disciplinary framework for addressing urban and socio-spatial implications of COVID-19 measures.

Answering complex questions like the ones presented in the preceding section requires responses underpinned by a commitment to trans-disciplinarity. It should be seen as a form of research involving co-operation among different parts of society, professionals, and academia ( Dunnin-Woyseth & Nielsen, 2004; Pohl & Hirsch Hadorn, 2008) in order to meet complex challenges in the context of COVID-19 spread and the resulting new normal and eventually the actual normal. Therefore, trans-disciplinarity is about blurring, then transcending, the boundaries of different disciplines. Starting from tangible, real-world problems, knowledge is developed, produced and reproduced based on a collaboration of people from different disciplinary backgrounds ( Doucet & Janssens, 2011). Its hybrid nature and non-linearity enables it to transcend and indeed incorporate any academic disciplinary structure. Adopting trans-disciplinary thinking at conceptual and critical analysis levels, a framework is developed through which responses to the questions are based on operationalization of concepts and theories derived from various disciplines as they relate to architecture and urbanism ( Figure 1), outlined as follows:

- Issues related to urban dynamics are delineated from the perspective of urban and human geography, urban design and planning, and transportation engineering.
- Questions that pertain to socio-spatial implications and urban space/ urban life dialectics stem from the field of environmental psychology.
- Deliberations about new environments that accommodate new living/working styles supervene from ethnographical and anthropological perspectives.

While the discussion does not fully address every single discipline shown in the diagram, all the perspectives and dimensions discussed have to do with researching and designing built environments.

## Urban dynamics during and post COVID-19

The notion that disease shapes and reshapes cities is acknowledged in many writings that have addressed various aspects of planners’ reactions to develop solutions to control transmission, including the ones referred to in this section. This includes the cholera outbreak in London in the 19th century, the Spanish flu in 1918 in New York and Mexico City, SARS in 2003, and, more recently, Ebola in West Africa in 2014. Interestingly, the discussions portray coronavirus as an unprecedented incident impacting urban peripheries, urban cores, global cities, and global networks. Perhaps what is completely new is that coronavirus is highly contagious, which makes it both locally and globally challenging. The nature of transformation in cities and urban dynamics can be understood through a critical analysis of two key areas, which are symbiotic: urban density and disease spread, and travel and transport and the associated global/local tensions.

### Urban density and disease spread

It is argued that infectious disease has a direct relationship with urban development and would result in significant impact on our understanding of urbanism. Hang (2020) suggests that the increasing density of cities has generated conditions for the rapid spread of viruses. By comparing the spread of SARS-CoV in 2003 and SARS-CoV-2 in 2019/20, it is evident that population density has a direct impact on the rapid spread. In the case of SARS-CoV within China, more than 5000 people were infected and more than 300 died over a period of eight months. Yet, in the case of SARS-CoV-2, more than 4000 people were infected over a period of seven weeks. This could be attributed to the fact that Chinese cities have developed into both dense and sprawling urban areas, with higher population density resulting from migration from rural to urban areas. Klaus (2020) interviewed Michele Acuto, professor of global urban politics in the School of Design at the University of Melbourne, and the question of density and density management were central to the discussion and were portrayed as the “ *long term survival in a pandemic world*.” Klaus (2020) supports this view and argues, “ *part of the history of urbanization is building and managing your way out of infectious diseases, such as cholera outbreaks in the middle of the 19th century*.”

Badger (2020) adopts an opposing view and argues that high density is not necessarily a public health issue. She evidences this by putting the case of Singapore and Hong Kong as urban environments that are dense or denser than New York, and how they were able to develop early testing and extensive tracing of coronavirus cases rather than extensive isolation. She advocates density for the multiple benefits it brings to cities’ unique cultural richness. These include facilitating mass transportation, creating walkable environments, enabling the masses to enjoy public spaces, supporting children’s needs through urban parks and playgrounds, limiting climate emissions, supporting personal and public safety, to name a few. It is increasingly acknowledged, following Sennett (2016) and Shenker (2020), that the future of cities will involve a renewed focus on developing architectural and urban solutions that enable people to socialise without higher densities and ‘sardines-like’ packing.

The work of Keil *et al.* (2020) suggests that there is a close relationship between urban development and new or re-emerging contagious diseases. Rapid means of transportation, the continuous expansion of urban sprawl, and connectivity between urban life and nature are factors that contribute to such a spread from urban peripheries to urban centres. However, patterns of disease emergence and spread within urbanisation would require in-depth empirical modelling and investigations of historical cases and juxtaposing these with the case of coronavirus spread.

The persistent question that urban design and planning experts will face in the future is about balancing conflicting value. On the one hand, densification and making cities more compact and concentrated for the environmentally and socially sustainable benefits compactness is viewed to bring. On the other hand, compartmentalisation and separation of populations through various measures including social distancing as a key tool being used to delay or stop virus spread. While compactness and density are important determinants for successful urban environments in cultural, social and environmental terms, current discussions suggest that the future development of cities will witness encounters between contested requisites including public health, climate, and socio-economic dynamics.

### Travel, transport, and the global/local tensions

In meeting the challenge of the COVID-19 spread, cities are adopting various strategies at a large scale. While cities are at the forefront of the response to the pandemic, they will likely see enduring transformation and permanent changes. While it is argued that cities and urbanism have been shaped by contagious disease for centuries, the global nature of COVID-19 is anticipated to bring significant changes to policies and the associated institutional and individual behaviours. In essence, these changes, while they have already started to alter the way people live and work, will alter how cities are planned and managed for decades to come. Null & Smith (2020) identified a number of areas that delineate various strategies restricting access to travel and questing for alternatives to public transit.

Locally and globally, measures for limiting travel are most apparent in the sense of how cities are now operating. In a short period of time travel restrictions, among other measures, have had negative impacts, including destroyed economies, unemployment, and a dramatic reduction in industrial production. However, some positive impacts are also witnessed in terms of a reduction in air pollution and carbon emissions. In the cases of Italy and China, NASA satellite data illustrate significant changes in air pollution levels ( NASA, 2020) and a 25% drop in carbon emissions as lockdown and restrictions started to show results ( Null & Smith, 2020).

Coupled with the reduction in train and bus operations in many cities around the world, local and global travel restrictions are expected to have significant implications on the what and how of urban planning in the future, given the established correlations between morality rates and urban air pollution, with positive impacts on the health of urban societies. However, these will need to be balanced in the context of climate change and sustainability. If the city is expanded rather than compacted, this must be associated with better connectivity through alternative forms of public transport. As people avoid crowds and movement is restricted, reports suggest that public transit use has taken a sharp decline ( Hawkins, 2020; Moovit, 2020; Nuki, 2020). The planning of future cities would be derived from enhanced policies that further support cycling and walking.

Contagious disease outbreaks are indeed global incidents, as evident in the spread of coronavirus from Wuhan to various cities around the world. Lockdown and quarantining of many mega and global cities is impacting the global condition at various scales, including globalised urban lifestyles. The rapid spread of disease takes place through infrastructures of globalisation, which includes global air travel links. Keil *et al.* (2020) argue that airports are often located at the peripheries of cities and urban areas and this raises questions about responsibility and accountability in terms of managing disease spread to the wider urban regions. In 2003, SARS impacted global centres of trade and commerce including Beijing, Hong Kong, Singapore, and Toronto. However, COVID-19 has severely impacted global cities in the global north, such as London and New York, and global infrastructure such as airport hubs, aviation networks, and places of industrial production.

An interesting phenomenon emerges out of the current discussion of the threat of COVID-19. There is a heavy focus on discussing repercussions of spread in universal terms and this reinforces existing inequalities ( Wilkinson, 2020). In particular, informal settlements and slums in the global south and the associated urban poverty do not seem to have enjoyed a sufficient share within current discussions and media reports. This could be attributed to the fact that these settlements are already on the margin and that communities are already underrepresented where crises are the norm and thus there is nothing new. Despite the significant lack of information, there is a risk that infection rates and transmission will be significantly higher than in planned cities and urban areas. To alleviate the negative consequences of these and introduce positive interventions in informal settlements in the long term, architects and planners would need to establish new lenses through which they can comprehend health and living conditions that generate relevant intervention strategies.

## Socio-spatial inferences of distancing measures

The spatial experience of people as individuals and communities is understood through wide-ranging concepts and notions, which stem from the field of environmental psychology and address buildings and urban environments. Geographical locations as they relate to the understanding of what constitutes home zone and home range conceptions, place attachment, personal space, and proximity to nature are important concepts that provide insights into social-spatial experiences.

### Geographical locations, home zone and home range

Geographical locations refer to the key places and areas that influence people’s perception of the city, such as living, working, shopping, and entertainment places or destinations. Derived from the theory of territoriality ‘home zone’ and ‘home range’ are constructs that represent areas which influence people’s mental image of important geographical locations. Establishing links between the physical environment and social behaviour, theorists and researchers have examined these insights in various contexts ( Abdel-Hadi *et al.* 2011; Altman, 1975; Rapoport, 2005; Salama *et al.* 2013). In this respect, it is important to discern how these constructs can be differentiated. Home zone signifies an environment with minor or no requirement for transportation. This includes locales that are accessed effortlessly through walking, thereby stimulating a sense of ownership and belonging among the residents within these locales. Irrespective of scale and size, home range denotes a more inclusive mental image of the entire residential environment, placing emphasis on the perceived geographical boundary such as that of residential neighbourhood or clusters of housing developments.

Within urban design and planning disciplines, a city is perceived as a fluid dynamic system, which involves material and non-material inputs and outputs that flow in, out, and within. This relates to the notion of geographical locations and represents, at a larger scale, a process of movement and mobility within the city and beyond. In historical writings ( Burgess, 1925; Sorokin, 1927) and contemporary debates ( Geyer & Kontuly, 1996) movement and urban mobility have been discussed from various perspectives. In the past, the focus was on social and behavioural factors. Recently however, the notion of networks, the spatial structure, and the people’s perception of both, were introduced.

Understanding urban mobility or movement patterns within the city will be crucial to conceiving interaction measures between geographical locations within the spatial structure, urban networks, and the associated operational requirements. The distribution of functions and uses, people’s movement patterns and the rhythm of commuting between geographical locations within the city, will all constitute important design and planning criteria that carefully consider health and the potential spread of disease within the city’s urban structure. Acting as imperatives, these considerations will enable lessening, or eliminating, the spread of viruses to various geographical locations, from the urban peripheries and around airports, to urban cores or residential neighbourhoods. This incudes in depth modelling of how far from and how long it takes to commute to the city core, from living areas to work areas, public places, entertainment places, and within residential neighbourhoods. Such modelling should also embed user types and age ranges, with a focus on vulnerable groups.

### Place attachment, personal space, and proxemics

Since social distancing measures are currently planned to last at least six months, it is believed that they will have various forms of impacts on both urban researchers and the public. Concepts and theories related to place attachment, personal space, and relationships between individuals and groups, as well as proximity to nature will need to be revisited.

Place attachment is highly influenced by an individual and his or her personal experiences ( Rollero & De Piccoli, 2010). It is multi-dimensional and cannot be explained through a cause and effect relationship since it depends on a reciprocal relationship between human behaviour and past and current experiences ( Giuliani, 2016; Scannell & Gifford, 2010). In essence, it relates to affect, cognition, and behaviour. Places of attachment include the home, neighbourhood, urban settings, and natural landscapes. Attachment to these places is typically measured through many qualities depending on the typology and use of place. These qualities include: aesthetics, heritage, family connection, recreation, therapeutic, biological diversity, wilderness, home, intrinsic, spiritual, economic, life-sustaining, learning, and future. Post pandemic place attachment conception would involve reweighing many of these qualities with more emphasis placed upon on qualities related to healthy, hygienic, sanitised, and healing environments. This may also lead to the rise of disaster psychology, which aims to examine the relationship between a city, urban area, or neighbourhood and their inhabitants’ attitudes and emotions in the context of detrimental incidents such as coronavirus spread and an increasing sense of personal safety and health.

The established canons of personal space and proximity relationships introduced in the mid 1960s ( Hall, 1966 and Hall & Hall, 1966) are critical to grasp in the context of social distancing measures. Personal space determines how people relate socially and psychologically and can be represented by an area (bubble) with an invisible boundary surrounding the person’s body into which intruders may not come. Such a bubble is carried everywhere one goes. Based on intensive empirical studies, Hall (1966) explained the relative distances between people depending on the relationships they have and classified them in four discrete distance ranges: intimate distance (1 to 46 cm), personal distance (46 to 122 cm), social distance (1.2 to 3.7 m), and public distance (3.7 to 7.6 m and more). With social distancing measures and the minimum allowance of 2 meters personal distance, the relative distance ranges would entirely change, especially if social distancing measures are viewed in the future as accepted standards.

Currently, there is a growing interest in designing healing environments. Ryan *et al.* (2014) argues that the surge of interest in creating spaces and places that support health and wellbeing is viewed as a renaissance in design thinking and the way in which buildings and cities are designed and built. Salingaros (2015) contends that biophilic design effectively eliminates stress and anxiety from the built environment and is achieved by maintaining thoughtful engagement with nature. Recently, researchers have been investigating the syntactic relationships between people and nature ( Asfour, 2019; Rice, 2019; To & Grierson, 2019). In such a quest, they attempt to demonstrate the role of architecture and urbanism in developing healthy and healing environments and how design can be informed to allow critical human/nature associations to prosper. Social distancing measures may encourage less association with people in urban settings and may give further rise to biophilic design trends.

### COVID-19 links to urban space/urban life dialectics

The ‘urban’ has been defined as “a place of encounter, assembly, simultaneity” ( Lefebvre, 1970:118). It is argued that there are two polar perspectives associated with the term, which stem from various disciplinary territories: urban form and urban life. While urban from is the sphere of urban designers, architects, urban planners, and transportation engineers, urban life is the territory of social scientists, human geographers, and sociologists. As argued by Susser (2002), urban form refers to the spatial concentration of populace within a specific area of land, limits to building and population densities, and certain qualities of buildings and spaces. Urban life epitomises the ‘collective,’ which refers to the diffusion of the system of values, attitudes, customs and behaviours of a specific group of people. Associations between urban form and urban life have been studied thoroughly ( Salama *et al.*, 2017a) through a sustained and consistent understanding that they cannot be discussed in isolation where urban form is shaped and influenced by urban life and urban life arises from urban form.

The urban place has been expressed in various studies as having contrasting attributes based on the characteristics of form and the nature of the activities taking place within that form. Positive attributes include diversity, tolerance, association, integration, personal network-formation ( Fischer, 1977) and frequent spontaneous interactions. While these qualities represent an ideal condition and confirm Lefebvre (1970) postulation, the urban place has been portrayed in negative attributes ( Halpern, 1995) that include anonymity, detachment, loneliness, formalised social control, segregation, isolation, fear, and mental illness. The COVID-19 social distancing measures will have an impact on the perception of some of these qualities, especially if these measures, in part or in whole, become the norm and part of the new normal. An example for this is that spontaneous interactions would become less (or non-) spontaneous while formalised social control will take place as a positive rather than a negative attribute.

The suggestion made by Walzer (1986) that public space “is where we share with strangers, people who aren’t our relatives, friends, or work associates” could entirely change given the cognizance of users regarding the risks associated with engaging with others they do not know. If social distancing measures become part of the collective psyche of societies, it could lead to a significant change in comprehending the needs in public spaces by revisiting the notions of social interaction, assembly, and simultaneity. Active engagement, which represents the direct experience a person has with a place and the people within it, would be limited or directed more towards passive engagement that involves meeting the need for encounter without becoming actively involved. This includes watching the passing scene rather than talking or doing.

Since the mid 1970s, urban theorists have conceived various triadic relationships that established a common understanding of places. Canter (1977) developed an understanding of the constituents of place, which include psychological conceptions, physical attributes, and actions and behaviours. Punter (1991) introduced mental image, form, and activity, while Mongomery (1998) discussed factors that generate a sense of place involving meaning, physical setting and activity. Post pandemic urban design would need to emphasise factors relevant to spatial proximity as it relates to health in order to limit the potential spread of viruses or reacting to people’s awareness of it. This could eventually lead to altering the triadic relationships, which were part of urban discourse for several decades.

## The spatiality of post pandemic emerging living/working styles

Social distancing guidelines coupled with operating in a post pandemic virtual world will instigate new living and working patterns, which will result in different spatial requirements and place standards. To understand the spatiality of these dynamics, it is crucial to relate to the body of knowledge developed within social sciences ( Adler *et al.*, 1987), which has significant implications on the notion of place as a human product involving human choices. Following Giddens (1984) and Pred (1984), these products and choices are based on factors relevant to how different elements of a society interact and a critical understanding of people, organisations, agencies, and the power that each element of a society has. This understanding can be further elaborated based on ethnographic and anthropological perspectives as they relate to the built environment ( Salama, 2011; Salama *et al.*, 2017b).

From an ethnographic perspective, the concept of life-mode ( Hojrup, 2003) offers insights into the understanding that human values are constrained by cultural-relational dialectics and are products of cultural life modes, which can be classified according to work styles: self-employed mode, wage-earner mode, and career-oriented mode. From an anthropological perspective, Douglas (1996) discusses a life style scheme that relates to attitudes rather than work. These attitudes can be envisaged as sub-cultures that include: competition and individualism, isolation and avoidance of social controls, equity and negotiation, and hierarchical communities. The two perspectives have direct spatial implications on how post pandemic emerging patterns of living and working will be spatially accommodated.

The spatial implications resulting from the two perspectives can be extrapolated when looking at various manifestations of the home environment and home choices ( Salama, 2011). In terms of work, the self-employed life mode is where means of production are owned and included within the house. This means that the home environment is conceived as an integrated living and working place where work time and spare time are not separated. The wage-earner life mode means that the home environment is regarded as either a primary place serving recreational purposes, or a place where important spare-time family or personal activities are undertaken. The career-oriented life mode means that the home environment reflects the personal progress, position, social status, and past and recent experiences. Additionally, the notion of sub-cultures determines housing preference based on social attitudes, especially isolation and avoidance of social control, and will manifest in post pandemic housing choices including preferred aspects related to the house integration within the neighbourhood, and the overall house size and image.

Whether work-based or status- and attitude-based, the spatial attributes of home and work environments post pandemic should be seen as a product of distancing measures and of operating in a virtual/digital world, which will instigate new living and working patterns. These implications will influence the existing housing stock and working places, which will require appropriation and adaptation and the new developments of home and work environments, requiring new standards and specifications.

## Outlook: the built environment and health in a post pandemic virtual world

**Figure 2.**
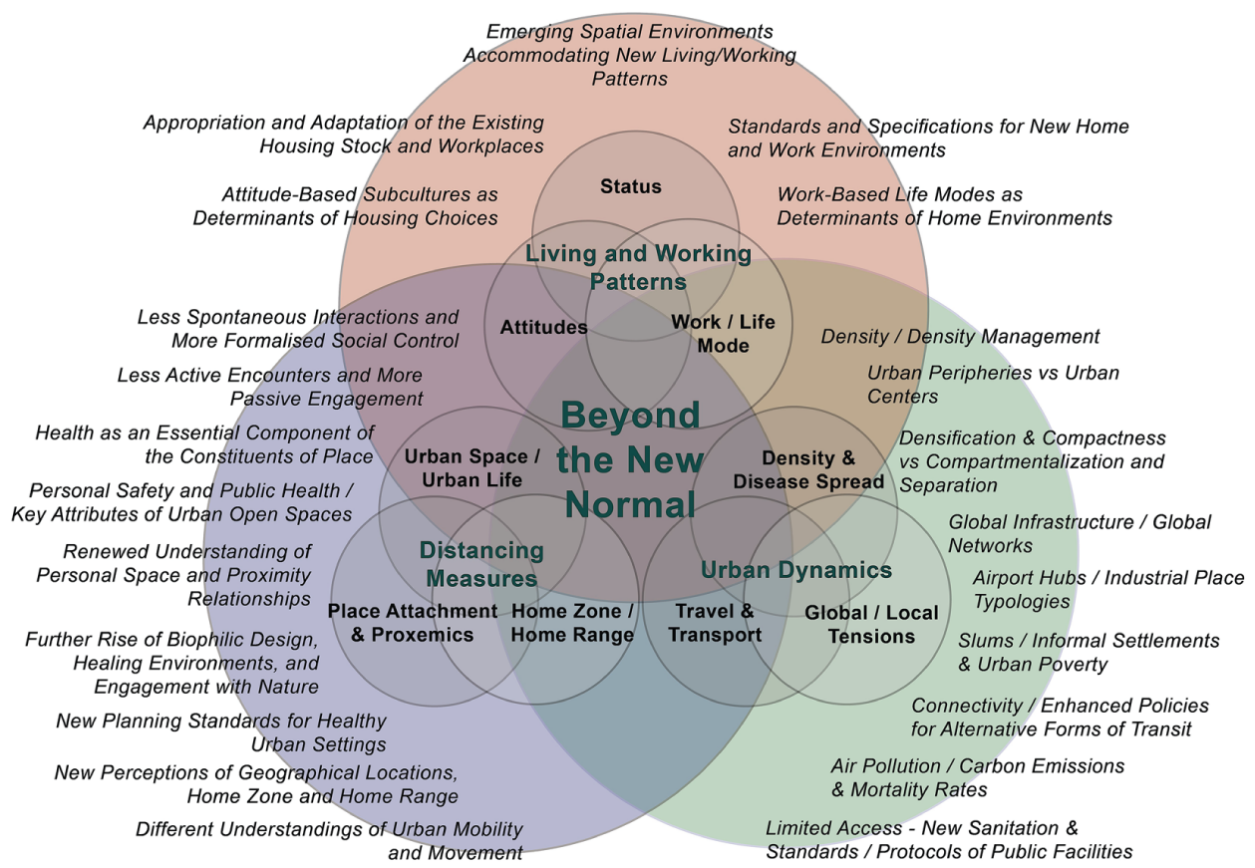
Post pandemic urban and socio-spatial implications, and potential areas impacting future education, research, and practice of architecture and urban design and planning.

The public health catastrophe caused by coronavirus and the rapid spread of COVID-19 disease has significant impacts on societies and cities around the world. The trans-disciplinary conceptual framework utilised for analysing urban and socio-spatial implications of COVID-19 measures reveals important insights into the factors that will impact future education, research, and practice of architecture and urban design and planning. These factors act as a base for potential contributions of architecture and urbanism as academic disciplines and professions to develop new insights into the impact of a pandemic on cities and urban environments and the socio-spatial implications of COVID-19 measures and guidelines. Immediate research areas were classified under three key areas: urban dynamics during and post COVID-19, socio-spatial implications of distancing measures, and the requirements of the new spatial environments to accommodate post pandemic emerging living and working patterns ( Figure 2). As the spread of COVID-19 has influenced individuals, communities, organisations, and governments, its impacts will be on every level and scale from global networks and infrastructure to global cities and urban regions, and from residential neighbourhoods and public spaces to home and work environments, and will continue for many years to come.

According to Forsyth (2020), “ *the current pandemic brings the question of designing for infectious diseases back to the forefront, however, and raises important questions for future research and practice*.” Therefore, the development of healthy environments must be central to architecture and urbanism in the future; despite that, health does seem to be absent within the education and practice of architecture and urban design and planning professions. Forsyth (2020) asserts this view and argues: “ *For the past decades, those looking at the intersections of planning, design, and public health have focused less on infectious diseases and more on chronic disease, hazards and disasters, and the vulnerable*.” Rice (2019) maintains that the design of the built environment is a determinant of health and thus there is an increasing need for greater synergy between architectural and urban education, research, and practice and public health.

The spread of the disease generated a condition, which is labelled as the new normal, resulting from social distancing measures, and is characterised by human detachment, isolation, and engagement in a virtual world, coupled with an emphasis on working from home through the utilisation of information and telecommunication technologies. The acceptability of the new normal as a result of attempting to limit the disease spread appears to be a catalyst for a future stable condition; the actual normal. While addressing health in a post pandemic virtual world, negative consequences emerge where many people around the world will be living and working in confined spaces, surrounded by gigantic cities and massive high-rise agglomerations.

The new normal – to be the actual normal – was foretold in the writings of theorists in architecture and urbanism. Manuel Castells in his book: The Rise of the Network Society: Economy, Society, and Culture (2000) developed a methodical theory of the information society, which is based on the overpowering impacts of information technology in a contemporary global world. His assumption that the global city is not necessarily a place but a process seems to manifest in the future stable condition. The visionary trilogy of the late William Mitchell is clear evidence that the actual normal represents the prospects of societies and cities.

The stable condition of the actual normal is palpable in the *City of Bits* ( Mitchell, 1995) where Mitchell examines architecture and urbanism in the context of the digital telecommunications revolution, the continuing miniaturization of electronics, the commodification of bits, and the growing domination of the digital over the physical. In the E-Topia (1999), Mitchell examines the way in which an electronically connected world will shape cities of the future and the associated urban relationships, with a focus on digital infrastructure and its implications for future daily lives. Mitchell asserts that we must extend the definitions of architecture and urban design to integrate virtual places as well as physical ones, and interconnection by means of telecommunication links as well as by pedestrian circulation and mechanized transportation systems. He proposes strategies for the creation of cities that not only will be sustainable but also will make economic, social, and cultural sense in an electronically interconnected and global world. While this conceptualisation would seem an imaginary future, it appears now as a representation of the stable condition of the actual normal. Mitchell’s book Me++: The Cyborg Self and the Networked City (2003) answers the question of how the transformation of wireless technology and the creation of an interconnected world are changing our environment and our lives. He argues a world governed less and less by boundaries and more and more by connections requires the reconstruction of our environment and our cities, and reconsideration of the ethical foundations of architecture, urbanism, and allied disciplines.

In a transition period called the new normal, which will eventually become a stable condition of the actual normal, societies, communities, and individuals appear to be at odds speculating the future. That future seems to have already arrived at our doorsteps, perhaps forcefully, perhaps arbitrarily, perhaps in a ‘shock and awe’ manner, but surely at the expense of disease, panic, mental illness, and death.

## Data availability

No data are associated with this article.
