# Supplementary material for: Coronavirus questions that will not go away: interrogating urban and socio-spatial implications of COVID-19 measures
Source: Emerald Open Res. 2020 Apr 16;2:14. doi: 10.35241/emeraldopenres.13561.1 (PMC7219560; doi:10.35241/emeraldopenres.13561.1)
Supplement: Peer review history

# Peer review: Reviewer response for version 1

**DOI:** 10.21956/emeraldopenres.14632.r26735

**Published:** 2020-04-23

**Authors:** James Brown

**Affiliations:** 1 School of Architecture, Umeå University, Umeå, Sweden

This is a timely yet well-structured and referenced provocation paper. It serves as a good foundation and literature review for the responsibilities of a profession and academic discipline.

 The text is well written, and there are no flaws in the syntax or grammar.

 I would recommend one minor improvement to the illustrations of the article. Figure 1 and figure 2 are legible, but dense with text and therefore difficult to read for those with different visual abilities. I would specifically recommend removing the watermark of the COVID-19 virus from figure 1 in subsequent revisions.

 I want to thank and commend the author for writing a meaningful contribution that will have much greater longevity than the vast number of “hot takes” which have been published in the recent weeks.

Is the topic of the opinion article discussed accurately in the context of the current literature?

Yes

Are arguments sufficiently supported by evidence from the published literature?

Yes

Are all factual statements correct and adequately supported by citations?

Yes

Are the conclusions drawn balanced and justified on the basis of the presented arguments?

Yes

Reviewer Expertise:

NA

I confirm that I have read this submission and believe that I have an appropriate level of expertise to confirm that it is of an acceptable scientific standard.

# Peer review: Reviewer response for version 1

**DOI:** 10.21956/emeraldopenres.14632.r26736

**Published:** 2020-04-22

**Authors:** Nabil Mohareb

**Affiliations:** 1 Faculty of Architecture-Design and Built Environment, Beirut Arab University, Beirut, Lebanon

Dr. Ashraf Salama is addressing an important set of questions related to the future consideration of the post pandemic COVID-19 effects on the urban and social-spatial spheres. These questions are genuine, but raise other sub-questions that need to be addressed as well in this paper in order to provide a sort of holistic overview of the current and post-pandemic situation.

 The author highlighted the unique behavior of COVID-19 in the section headed ‘urban dynamics during post COVID-19’, which is based on its high level of contagion, and related its spread to urban densities and travel. Even densities, which the author has addressed as one of the causes of the disease spreading, might not be equally important factors in all cases, if the number of cases were reviewed in developing countries with high density cities. Therefore, I would suggest addressing the following question: is it the human behavior when using space that should be questioned and changed in the post-pandemic era or the urban design and planning theories underlying the design of that space?

 The author has highlighted previous pandemic diseases that hit specific areas, such as the cholera outbreak in London, Spanish flu in New York, SARS and others, so the question arises: did these pandemics alter the city/urban spaces/ planning of these cities?; did they have a direct effect on the urban and social-spatial design?; or did they change the behavior of the inhabitants? I think these points should be addressed by measuring the impact of previous pandemics or disasters on cities to forecast the possible impact of COVID-19 on urban and socio-spatial issues in the future.

 Clearly, COVID-19 has hit heavily countries and cities with varying health and urban space quality levels, in both the Global North and South, and there have been more cases in the well-designed, healthier Global North cities than in those in the Global South. I think that it is important to address this point in the paper, which states as its main question: high living standard cities or cities with poor design qualities? In other words, does this paper address architecture and urban design/planning in general or in a specific location?

 Finally, defining the term ‘the new normal’ is essential: is this a global term or will there be a specific ‘new normal’ for each place, with socio-cultural and economic differences between different areas?

 In general, I would like to thank the author for his efforts in writing this paper.

Is the topic of the opinion article discussed accurately in the context of the current literature?

Yes

Are arguments sufficiently supported by evidence from the published literature?

Yes

Are all factual statements correct and adequately supported by citations?

Yes

Are the conclusions drawn balanced and justified on the basis of the presented arguments?

Yes

Reviewer Expertise:

NA

I confirm that I have read this submission and believe that I have an appropriate level of expertise to confirm that it is of an acceptable scientific standard.

# Peer review: Reviewer response for version 1

**DOI:** 10.21956/emeraldopenres.14632.r26737

**Published:** 2020-04-21

**Authors:** Karine Dupre

**Affiliations:** 1 Department of Architecture, Griffith University, Gold Coast, Qld, Australia

This is a very timely article, laying out the current opinions on the post COVID period and relevantly questioning the role of architecture and urban planning and design in the process.

 Few comments

 p5: ‘The notion that disease shapes and reshapes cities is acknowledged in many writings that have addressed various aspects of planners’ reactions to develop solutions to control transmission, including the ones referred to in this section. ‘There is a full historiography of the colonial enterprise shaping cities to control diseases and germs (e.g. Nezar Alsayyad, Forms of Dominance... 1992). Maybe few should be cited here to give more weight to the argument? (besides Cerda’ Example or Haussmann’s grandes avenues to instaure hygienist principles and fight cholera outbreaks and tuberculosis transmission)

 p5: ‘Perhaps what is completely new is that coronavirus is highly contagious, which makes it both locally and globally challenging.‘ However the previously mentioned cholera, Spanish flu, SARS and Ebola are also highly contagious. Maybe another new-ness is rather the world travel context which has perhaps reached a very high level and specifically with Chinese residents being allowed to i) travel and ii) travel on their own (also both rights have been slightly altered since 1-2 years) since the late 2000s, representing nearly 18% of the global population ^1^. 

 p8: ‘The spatiality of post pandemic emerging living/ working styles Social distancing guidelines coupled with operating in a post pandemic virtual world will instigate new living and working patterns, which will result in different spatial requirements and place standards. (...)’

 In this section, could it be possible to add some nuances to some sentences (as the one above for instance)? We understand the text is somehow simplified to convey the message but as such lacks to represent fully the diversity of cultures and lifestyles that are currently existing and the role of architecture and urban planning and design to sustain them.

  Interesting readings such as Friedmann, J. (2007), “Reflections on place and place-making in the cities of China”, International Journal of Urban and Regional Research, Vol. 31 No. 2, pp. 257-279 ^2^ and Karine Dupre, (2018) "Trends and gaps in place-making in the context of urban development and tourism: 25 years of literature review", Journal of Place Management and Development ^3^, (specifically section on place-making and globalisation) might also contribute to highlight the significance of smaller scale places, whether they be real or virtual.

 Many thanks for an interesting reading!

Is the topic of the opinion article discussed accurately in the context of the current literature?

Yes

Are arguments sufficiently supported by evidence from the published literature?

Yes

Are all factual statements correct and adequately supported by citations?

Yes

Are the conclusions drawn balanced and justified on the basis of the presented arguments?

Yes

Reviewer Expertise:

NA

I confirm that I have read this submission and believe that I have an appropriate level of expertise to confirm that it is of an acceptable scientific standard.

## References

[1] : We depend so much more on Chinese travellers now. That makes the impact of this coronavirus novel. Reference source

[2] : Reflections on Place and Place-making in the Cities of China. *International Journal of Urban and Regional Research*.2007;31(2) : 10.1111/j.1468-2427.2007.00726.x 257-279 10.1111/j.1468-2427.2007.00726.x

[3] : Trends and gaps in place-making in the context of urban development and tourism. *Journal of Place Management and Development*.2019;12(1) : 10.1108/JPMD-07-2017-0072 102-120 10.1108/JPMD-07-2017-0072
